# The Chinese Adolescent Cohort Study: Design, Implementation, and Major Findings

**DOI:** 10.3389/fnut.2021.747088

**Published:** 2021-11-05

**Authors:** Ruonan Duan, Xiaoyu Wang, Shufang Shan, Li Zhao, Jingyuan Xiong, Lars Libuda, Guo Cheng

**Affiliations:** ^1^Laboratory of Molecular Translational Medicine, Key Laboratory of Birth Defects and Related Diseases of Women and Children (Sichuan University), Department of Pediatrics, Center for Translational Medicine, Ministry of Education, West China Second University Hospital, Sichuan University, Chengdu, China; ^2^Healthy Food Evaluation Research Center, Sichuan University, Chengdu, China; ^3^Faculty of Natural Sciences, Institute of Nutrition, Consumption and Health, Nutrition Sciences, Paderborn University, Paderborn, Germany

**Keywords:** cohort study, nutrition, growth, obesity, puberty, children, China

## Abstract

The importance of diet quality on children's growth is being increasingly recognized. The Chinese Adolescent Cohort (CAC) is a longitudinal cohort study to comprehensively investigate the health impacts of nutritional factors on child growth. From 2013 to 2018, 6,967 children aged 6–8 years have been recruited from 23 primary schools in Sichuan, Guizhou, and Chongqing, which have been planned to be followed up annually until their age of 15 years. Regular assessments included the measurement of height, weight, waist circumference, and skinfold thicknesses; pubertal development was examined by trained investigators according to Tanner stages; dietary intake was obtained by three 24-h recalls and food frequency questionnaire; validated questionnaires were used to estimate socio-demographic characteristics, physical activity, and sedentary behaviors. Findings from the CAC baseline and the first follow-up data suggested that higher protein intake among girls and unhealthy eating habits among children might increase the risk for childhood obesity. Also, higher intakes of grain and meat and lower overall diet quality and intakes of dietary fiber and tuber might be associated with advanced pubertal development. Those results indicated that the CAC study could contribute to the development of strategies for optimizing Chinese children's health.

## Introduction

The impacts of diet quality on children's growth have been investigated for decades. Evidence from some recent studies has suggested that dietary intake during childhood may have long-term impacts on health, such as obesity in adulthood (1). Such findings have consistently emphasized the importance of diet quality in early life. In recent years, the prevalence of childhood obesity has risen alarmingly in China (2, 3). Meanwhile, a secular trend of earlier puberty onset has been observed in both Chinese boys (4) and girls (5). Both an earlier onset of puberty and childhood obesity have been considered to be risk factors for chronic diseases in later life (6–8). Although previous studies have revealed the relationship between diet quality and growth during childhood, little is known about the mechanisms underlying the impacts of diet quality on the development of obesity or early pubertal development among Chinese children. Since dietary habits in China are in a transition period (9), knowledge on dietary determinants that might pronounce the trends in obesity and puberty onset is of major public health relevance. Also, whether childhood obesity may contribute to early onset of puberty remains controversial (10). Thus, high-quality longitudinal cohort studies with prospectively collected information on childhood exposures are greatly needed in China.

To date, although an array of child cohorts have been established worldwide to address the effects of childhood exposures on subsequent health outcomes (11–18), only a few of them have focused on childhood nutrition and pubertal development. For example, the DOrtmund Nutritional and Anthropometric Longitudinally Designed (DONALD) Study (11), a German cohort, has collected detailed data on diet, anthropometry, and endocrinology of children from infancy to adolescence or early adulthood. However, the sample size was relatively small and non-representative. The 1993 Pelotas (Brazil) Birth Cohort has covered information on lifestyles (e.g., dietary patterns, physical activity) and child's growth (e.g., anthropometry, age at menarche) during the follow-up visits at 4–11 years (18, 19). However, the indicator of puberty timing was limited to menarche, which only represents a relatively late stage of pubertal development in girls (18). The China-Anhui Birth Cohort study (17) is another example of birth cohorts, which has focused on environmental exposures for children's development (including puberty timing), with limited information collected on childhood nutrition. To our knowledge, few large-scale child cohort studies addressing nutrition on children's growth existed in Southwest China. To fill this gap, the Chinese Adolescent Cohort (CAC) study aims to provide comprehensive and reliable data that can be used to investigate nutritional and lifestyle determinants of children's growth, with emphasis on overweight/obesity and pubertal development.

## Materials and Methods

### Design and Study Population

**Figure 1:**
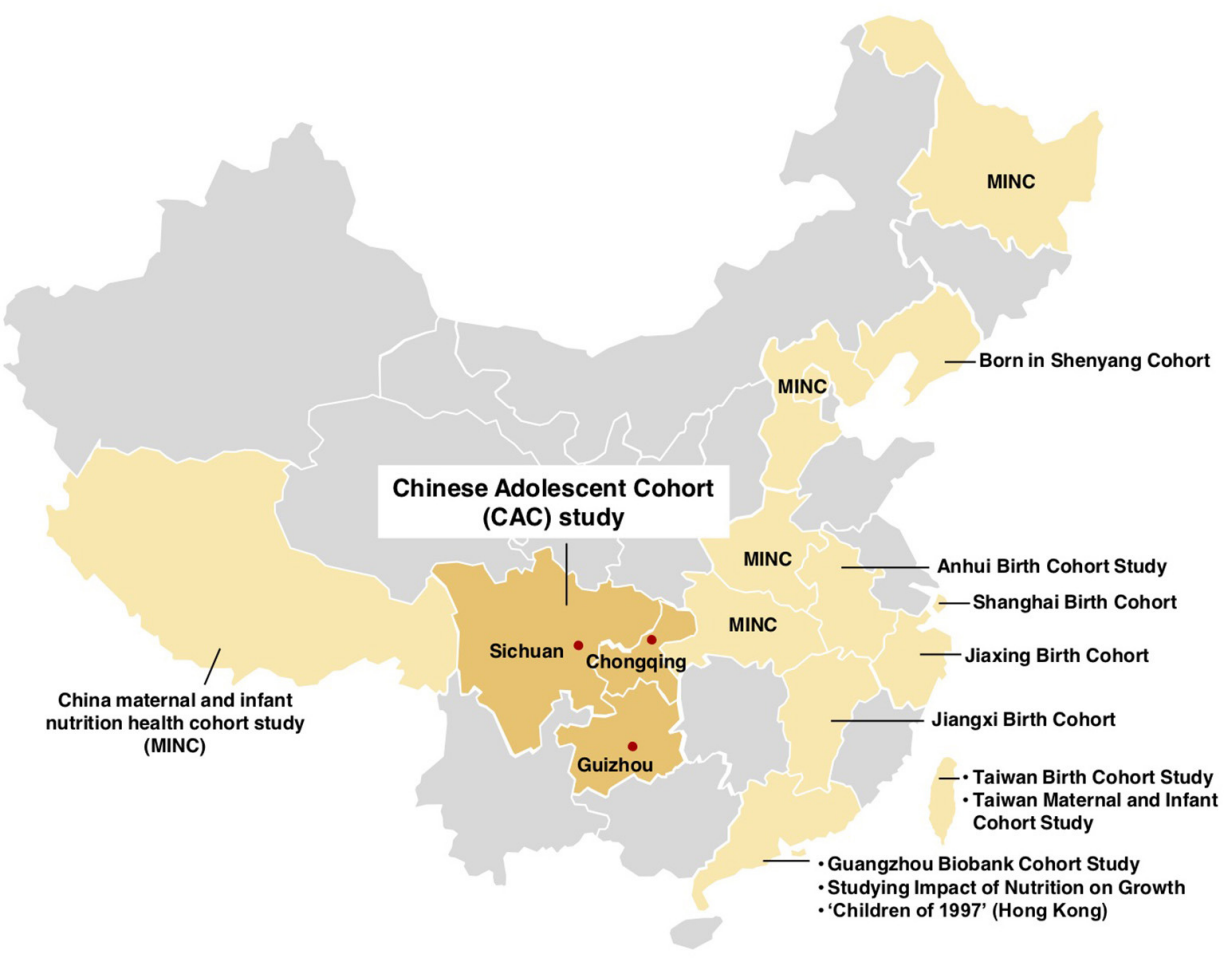
Location of the Chinese Adolescent Cohort study and other child cohorts in China.

The CAC study has been designed in Sichuan Province, Guizhou Province, and Chongqing Municipality, which are located in Southwest China, a large area of 2.5 million square kilometers with a population of 199 million (including about 2.5 million children aged 6–8 years) (Figure 1). The CAC is a longitudinal cohort collecting detailed data on diet, lifestyle behaviors, and children's growth across the entire pubertal period. Subjects were recruited at their age of 6–8, and would be followed up until their age of 15. Regular assessments include the measurement of anthropometry and puberty status every year, and the recording of diet, lifestyle behaviors, and parental characteristics every 2 years.

The recruitment of study participants started in January 2013. The starting study sample included children recruited from a baseline cross-sectional study (i.e., Diet Quality during Childhood), which aimed to collect information on the diet, anthropometry, and lifestyle of children in Southwest China (20). A sampling design stratified by urban and rural locations was used to obtain the study sample. Twenty-three study sites (10 primary schools in urban areas and 13 primary schools in rural areas) were included as of December 2018. The eligible participants were children aged 6–8 years, cooperative and voluntary at the time of recruitment, in the selected primary schools. Those who were multiple births, preterm (<37 weeks of gestation), or late term (>42 weeks of gestation), as well as those with birth weight lower than 2,500 g or major organ diseases (e.g., heart, liver, or kidney disease), were excluded. This study was approved by the Ethics Committee of Sichuan University. All methods were performed in accordance with the relevant guidelines and regulations. All parents gave written informed consent and all children gave assent (21).

### Follow-Up Assessments

**Figure 2:**
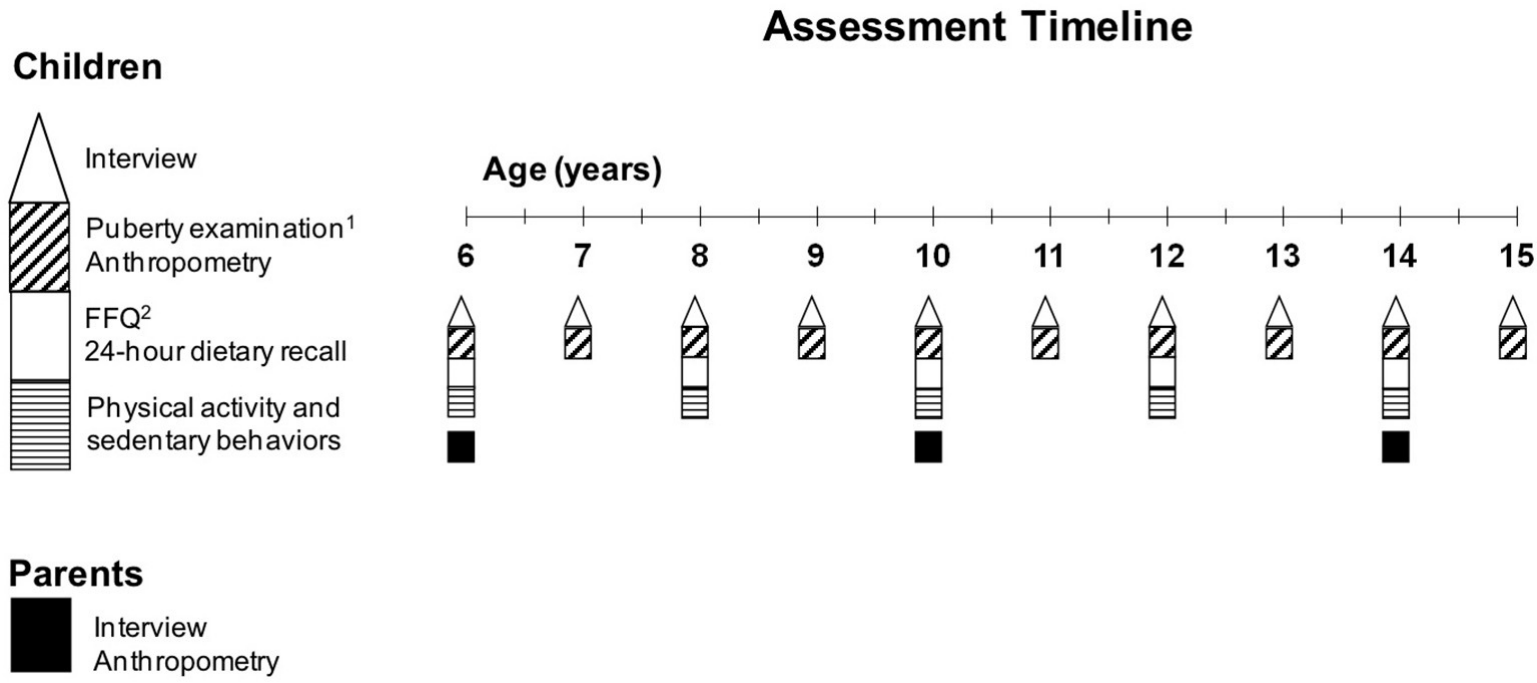
Assessment timeline of the Chinese Adolescent Cohort study. ^1^Puberty examination includes breast development and pubic hair development examinations in girls and testicular development and pubic hair development examinations in boys. In addition, children are asked whether menarche (girls), spermarche or voice break (boys) has already occurred and the respective month and year is recorded. ^2^ Food frequency questionnaires.

**Figure 3:**
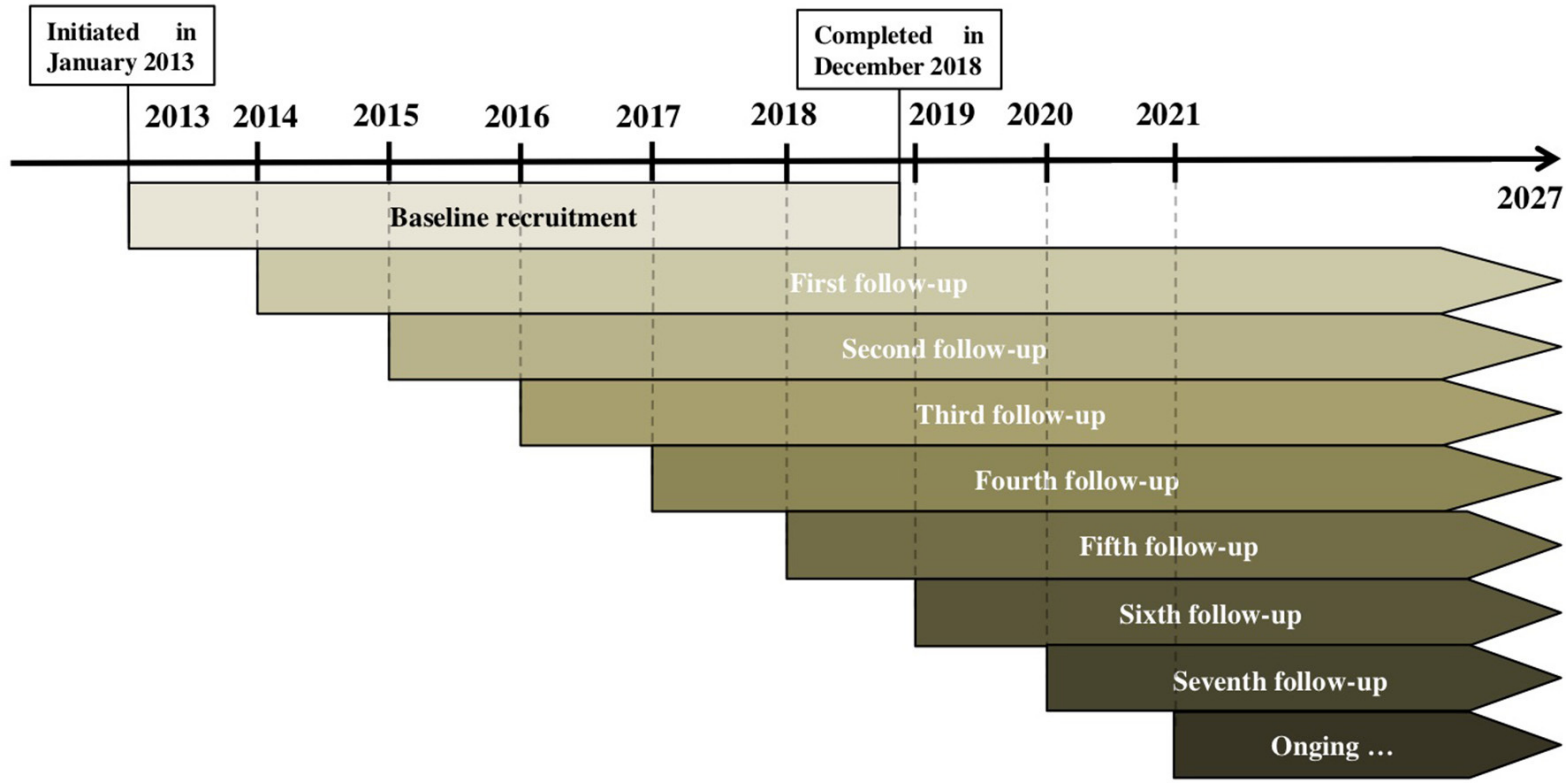
Follow-up periods of the Chinese Adolescent Cohort study. Each participant will be followed up for 8–10 times (from age of 6–8 years to 15 years). Up to now, seven follow-up assessments have been completed for participants recruited in 2013, and the eighth follow-up survey is ongoing in 2021. Follow-ups of children recruited from 2014 to 2018 have been carried out six times, five times, four times, three times and twice, respectively.

Children in the CAC study have been planned to be followed up from 6–8 years until the age of 15. Thus, the entire pubertal period would be captured (i.e., before, during, and after the pubertal period). In the first examination, information on socio-demographic issues, dietary intake and eating behaviors, physical activity and sedentary behaviors, and anthropometry and pubertal development were obtained. Follow-up assessments of anthropometry and puberty status were conducted every year, while data of dietary intake and physical activity were collected every other year. The study protocol was summarized in an assessment timeline (Figure 2). By the time of conducting this study, seven follow-up assessments have been completed for participants recruited in 2013, with the eighth follow-up survey ongoing in 2021. Follow-ups of children recruited in 2014 to 2018 have been carried out for six times, five times, four times, three times and twice, respectively (Figure 3).

### Data Collection

The CAC study assessment procedures were based on several modules: dietary intake and eating behaviors, physical activity and sedentary behaviors, anthropometric measurements, pubertal development examination, and some additional information asked in face-to-face interviews by trained investigators using a parent questionnaire (Table 1).

**Table 1:** Variables and instruments to be used in the Chinese Adolescent Cohort (CAC) study.

**Type of Exposure Data**	**Instruments**	**Variables for Exposure/Outcome**
Dietary data	Three 24-h recalls and food frequency questionnaire	Energy intake, macro- and micro-nutrients, food and food groups
Physical activity	Physical activity questionnaire	Met^a^-hours, time spent on sedentary behaviors per day
Anthropometry	Ultrasonic Weight and Height Instrument, non-elastic tape, Holtain caliper	Height, weight, waist circumference, skinfold thickness
Pubertal development	Prader orchidometer and questionnaire	Pubertal maturation for breast (girls) and pubic hair (girls and boys) stages, testicular volume, menarche (girls), spermarche or voice break (boys)
Children's birth characteristics	Questionnaire	Gestational age, birth weight, birth length, breastfeeding and complementary feeding practices
Socio-demographics	Questionnaire	Residence, household income, family size, passive smoking, parental age, parental occupation and education levels, parental height and weight, mother's menarche age

a *MET, metabolic equivalent*.

### Dietary Intake and Eating Behaviors

Participants were interviewed with respect to their diet (i.e., one food frequency questionnaire covering the consumption over the past 12 months, and three 24-h dietary recalls). The dietary assessment in young children was challenging, as they may not be able to provide relevant information by themselves. In the CAC study, children were asked to recall their consumption of all foods and beverages only when they were aged 9 years or older. When they were younger than nine at the time of interviewing, children provided only the dietary intake information from school by themselves, with the information on food consumption by parents at home.

Three 24-h recalls were used to collect dietary intake data by trained investigators. Details on recipes and brands of all food items reported were inquired. Food models for foods, standard tableware including bowls, plates, and glasses were provided to improve the accuracy of the portion-size estimates. In addition, children have been given a photo book, which contained photos of snacks and beverages and pictures of the commonly used commercial packaging (e.g., one carton) to improve the diet recall. The dietary intake data from 24-h recalls were converted into energy and nutrient intake data, using the continuously updated in-house nutrient database that reflected the China Food Composition (22) in NCCW software (version 11.0, 2014).

In addition, children's consumption of foods and food groups over the past year was collected with a validated food frequency questionnaire (23), which included 53 foods or food groups that were most representative of local consumption and ultimately grouped into 17 categories: whole grains, refined grains, tubers, vegetables, fruits, nuts, meat, fish and shrimp, animal viscera, eggs, dairy and dairy products, soybeans and its products, fried foods, sugary snacks, sugar-sweetened beverages, fruit juice, and dietary supplements. Participants were asked to report the frequency and amounts of their consumption for each food item.

For eating behaviors relevant to diet quality of children, participants were asked about whether or not they often ate breakfast (at least 5 days per week) or had dinner with parents/grandparents regularly (at least 5 days per week), with the corresponding time and places of breakfast, lunch, dinner and snacks recorded.

### Physical Activity and Sedentary Behaviors

A validated physical activity questionnaire (PAQ) (24) was used to collect information on the frequency, duration, and type of physical activities inside and outside school settings and leisure-time activities. The PAQ included a checklist of 38 items, e.g., walking, running, climbing stairs, ball games, dancing, and so on. In addition, participants were asked to report the usual time spent on sedentary behaviors (i.e., watching television, using computers, using smart phones, and doing homework) on weekdays and weekends.

### Anthropometric Measurements

Children in the CAC study have been undergoing the anthropometric measurement every year, which were performed by investigators who have been trained according to standard procedures, with the subjects dressed lightly and barefoot. An Ultrasonic Weight and Height Instrument (DHM-30, Dingheng Ltd, Zhengzhou, China) was used to assess the standing height to the nearest 0.1 cm and weight to the nearest 0.1 kg. Waist circumference at the umbilicus was measured to the nearest 0.1 cm by a calibrated measuring tape. In addition, skinfold thickness of biceps, triceps, anterior superior iliac spines, and subscapular angle sites were measured on the right side of the body to the nearest 0.1 mm using a Holtain caliper (Holtain Ltd, Crymych, UK). All anthropometric measurements were performed twice with the average calculated for each participant.

### Pubertal Development Examinations

During the annual physical examination for children, pubertal maturation for breast (girls) and pubic hair (girls and boys) stages was assessed by trained investigators according to the standardized criteria of Tanner stages (25). Testicular volume was assessed by comparative palpation with the Prader orchidometer. If the testicular volumes of the two testes were not equal, the volume of the larger one was recorded. Testicular volume <1 mL was recorded as 1 mL. In addition, children were asked at each examination about whether or not menarche (girls), spermarche, or voice break (boys) had already occurred, with the corresponding month and year information recorded.

### Additional Information

Information on children's birth characteristics and feeding practices (i.e., gestational age, birth weight, birth length, exclusive breastfeeding duration, and timing of complementary feeding) and socio-demographic data (i.e., place of residence, household income, family size, parental age, and parental occupation and education levels) were provided by parents. In addition, parents' height and weight were self-reported, and information on mother's menarche age was also collected.

### Quality Control

To ensure the accurate and standard measurement throughout the CAC study, several quality assurance measures have been undertaken. Prior to study implementation, a 3-day training was conducted in study offices at each site. A training manual describing all of the standardized procedures in details was developed, and all instruments for data collection (i.e., Ultrasonic Weight and Height Instrument, non-elastic tape, and Holtain caliper) were calibrated. During the study, data checks were conducted for completeness and accuracy immediately after questionnaires were completed. For data entry and management, double entries were carried out and corrections were made as needed. In addition, a pencil box or a notebook was delivered to each participant to encourage him/her to complete the interview.

## Results

Between January 2013 and December 2018, 7,119 children from 23 primary schools in Southwest China have been invited for the baseline data collection. Among them, 152 children refused to enroll due to parental disapproval. They did not differ in their basic characteristics (e.g., age and gender) from those who have participated. However, non-respondents were more likely to have lower parental education level and to live in rural areas (data not shown). Thanks to the efforts of the participating primary schools in assisting with data collection, 6,967 children completed the baseline questionnaire (2,320, 912, 926, 921, 986, and 902 in 2013–2018, respectively). Their baseline characteristics were shown in Table 2. Of the 6,967 participants, 52.6% were girls, with an average age of 7.8 years and moderate socioeconomic status. Those who agreed to participate were representative of the general children population in terms of age and parental education level and socioeconomic status (26).

**Table 2:** Baseline characteristics of 6,967 children in the Chinese Adolescent Cohort (CAC) study.

**Characteristics**	**Value**
Age, y	7.8 (0.6)
Female, %	52.6
Urban residence, %	52.1
Birth weight, kg	3.3 (0.5)
Breastfeeding for 6 months, %	69.2
Complementary feeding from 6th month, %	31.7
Family size, n	4 (1)
High family income level^a^, %	23.9
High maternal education level^b^, %	21.2
High paternal education level^b^, %	26.3
Weight, kg	24.4 (4.5)
Height, cm	121.3 (6.0)
BMI-SDS^c^	0.2 (1.0)
Percent body fat, %	16.2 (12.9, 23.1)
Overweight (including obesity), %	19.9
Energy intake, MJ/d	7.5 (6.4, 8.9)
Protein, % of energy	16.5 (14.3, 17.8)
Fat, % of energy	26.3 (21.3, 31.9)
Carbohydrate, % of energy	57.2 (52.8, 65.2)
MVPA energy expenditure^d^, MJ/day	0.5 (0.2)
Sedentary behaviors, h/d	2.1 (0.6)

a *Average family income more than 35,000 Yuan every year*.

b *At least 12 years of school education*.

c *BMI-SDS calculated according to the Chinese reference curves*.

d *MVPA energy expenditure, energy expended on moderate-to-vigorous physical activities (MJ/day)*.

Figure 3 showed the numbers of participants recruited and followed up until April 2021. A total of 1,262 children (18.1%) were lost to first follow-up due to several reasons (e.g., refusal to complete time-consuming questionnaires, moving away from the study area, parental disapproval). Children who were lost to follow-up were generally younger, with lower parental education and family income levels, spending more time on sedentary behaviors than their counterparts who retained in the study (Table 3).

**Table 3:** Comparison of basic characteristics between children who remained in the cohort for at least once follow-up and those lost to follow-ups.

**Characteristics**	**Children who were followed up**	**Children lost to follow-up**	***P* value**
N, %	5,705 (81.9)	1,262 (18.1)	
Age, y	7.9 (0.5)	7.5 (0.7)	0.04
Female, %	52.9	51.6	0.7
Urban, %	52.2	51.8	0.6
Birth weight, kg	3.3 (0.5)	3.2 (0.6)	0.8
Breastfeeding for 6 months, %	69.2	65.1	0.6
Complementary feeding from 6th month, %	32.1	29.9	0.08
Family size, n	4 (1)	5 (1)	0.07
High family income level^a^, %	26.3	12.9	0.04
High maternal education level^b^, %	22.2	16.5	0.03
High paternal education level^b^, %	28.1	18.3	0.03
Weight, kg	24.2 (4.3)	24.5 (4.6)	0.1
Height, cm	121.6 (6.2)	120.7 (5.9)	0.2
BMI-SDS^c^	0.5 (0.8)	0.5 (1.1)	0.9
Percent body fat, %	16.3 (13.1, 23.2)	15.8 (12.7, 22.9)	0.2
Energy intake, MJ/d	7.4 (6.3, 8.8)	7.6 (6.5, 9.2)	0.3
Protein, % of energy	16.9 (14.6, 18.1)	15.8 (14.1, 17.3)	0.06
Fat, % of energy	26.3 (21.1, 32.2)	26.4 (21.4, 31.8)	0.08
Carbohydrate, % of energy	56.8 (53.3, 66.8)	57.8 (52.8, 65.2)	0.07
MVPA energy expenditure^d^, MJ/day	0.7 (0.3)	0.4 (0.5)	0.051
Sedentary behaviors, h/d	1.7 (0.5)	2.3 (0.7)	0.04

a *Average family income more than 35,000 Yuan every year*.

b *At least 12 years of school education*.

c *BMI-SDS calculated according to the Chinese reference curves*.

d *MVPA energy expenditure, energy expended on moderate-to-vigorous physical activities (MJ/day)*.

As the follow-up assessments of CAC study are still ongoing, it is still at an early stage with data analysis in progress. To date, more than 10 peer-reviewed publications based on the CAC baseline and the first follow-up data have been published, covering different aspects of exposures and health outcomes. A summary of those findings was presented in Table 4, with the key findings representing multidisciplinary nature of CAC data collection presented below.

**Table 4:** Key published findings from the baseline and the first follow-up data of Chinese Adolescent Cohort (CAC) study.

**Key variables**	**Main findings**
**Diet quality**	
Development of dietary index	The Chinese Children Dietary Index was successfully developed and used to assess overall diet quality among Chinese children (21).
Diet quality and socio-demographic factors	Diet quality of children was associated with age negatively and family size positively (21).
**Obesity**	
Prevalence of overweight/obesity	Prevalence of overweight and obesity was relatively high among children in Southwest China (27).
Obesity and dietary energy density	Dietary energy density was not associated with body composition of children in Southwest China (20).
Obesity and protein intake	Girls, but not boys, with higher dietary protein intake might have higher body fat mass (28).
Obesity and dietary calcium/dairy	Boys with higher dietary calcium intake had the lowest prevalence of overweight (29).
Obesity and dietary pattern	Dietary pattern consisting of beans, potatoes, whole grains and dairy was associated with a lower risk of overweight and obesity (30).
Obesity and eating behaviors	Poor eating behaviors, characterized by sugar-sweetened beverages, not eating breakfast regularly and not having dinner with parents, are associated with child obesity (31).
Obesity and socioeconomic status	Urban girls living in relatively affluent circumstances were at greatest risk for being overweight (32).
**Pubertal development**	
Status of pubertal development	Genital/breast development among children in Southwest China starts later, while spermarche/menarche is earlier (33).
Pubertal development and dietary fiber	Dietary fiber intake, especially fruit fiber, is lower in children with earlier pubertal development (34).
Pubertal development and grain/tubers	Grain intake was positively associated with pubertal development among boys. Both grain and tubers intake were negatively associated with pubertal development among girls (35).
Pubertal development and meat and dairy	Level of pubertal development was positively associated with meat intake among boys, and negatively associated with dairy intake among girls (36).
Pubertal development and overall diet quality	Girls and boys with higher diet quality were more likely to enter their puberty later than their counterparts with lower diet quality, which were independent of pre-pubertal body fat (37).
Pubertal development and eating behaviors	Level of pubertal development was positively associated the frequency of snacks and sugar-sweetened beverages consumption among both boys and girls (38).
Pubertal development and socio-demographic factors	Pubertal development is related to parental educational level and occupation (33).
**Sedentary behaviors**	
Sedentary behaviors and socio-demographic factors	Time spent on watching TV was negatively associated with paternal or maternal education level. Time spent on using computers was positively associated with family income (39).
Sedentary behaviors and childhood body composition	Sedentary behaviors were positively and independently related to fat mass among Chinese children, and were more pronounced in girls (40).

### Diet Quality

According to the Chinese Dietary Guidelines and Dietary Reference Intakes, the Chinese Children Dietary Index (CCDI) was developed to assess the overall diet quality among the Chinese children. It showed the need to improve the diet quality among children in Southwest China, especially in boys, because their consumption of grains and meat were excessive with inadequate consumption of soybeans, fish, shrimp, eggs, and dietary fiber. In addition, socioeconomic factors were identified to be correlated with the CCDI score, which revealed an impact of paternal, rather than maternal, education level on children's diet quality. A positive association between the CCDI score and family size was further observed (21).

### Obesity

We found that the prevalence of overweight/obesity among children in Southwest China was relatively high: 10.7 and 6.5% of CAC children were observed as overweight and obesity, respectively, compared to the overall situation that the prevalence of childhood overweight and obesity has risen from 4.5 and 2.3% in 2002 to just 9.6 and 6.4% in 2012, respectively (27).

As a modifiable factor, dietary intake has played an important role in the development of childhood overweight/obesity. In the CAC study, girls, but not boys, with higher dietary protein intake may have higher body fat mass, which, further modified by paternal occupation, was observed only in girls with part-time working fathers (28). Also, children with healthy dietary patterns (i.e., beans, potatoes, whole grains, dairy) were at lower risk for overweight/obesity (OR = 0.59, 95% CI [0.39, 0.90]) (30). Unhealthy eating habits, characterized by sugar-sweetened beverages, not having breakfast, and not having dinner with parents/grandparents, have increased the risk for childhood obesity (31).

### Pubertal Development

Compared with children in western countries and in eastern and central cities of China, genital/breast development among children in Southwest China was later (33). Low dietary fiber (especially fruit fiber) (34) and tuber intake (35), and high grain (35) and meat intake (36) may be associated with advanced pubertal development, which were more pronounced in girls. Additionally, based on data from 3,983 CAC study participants (1,752 girls and 2,231 boys) with at least 2 follow-up assessments by the end of 2019, the prospective relevance of diet quality on puberty timing were examined. We found that both girls and boys with higher diet quality were more likely to enter their puberty later than their counterparts with lower diet quality, which were independent of pre-pubertal body fat (37).

### Sedentary Behaviors

In the CAC study, children with high paternal or maternal education level tended to spend less time on watching TV, while those living in high-income families seemed to spend more time on computer use (39). Furthermore, sedentary behaviors were positively and independently related to fat mass among children in Southwest China, which were also more pronounced in girls (40).

## Discussion

This study described the design and implementation procedures of the CAC cohort, and summarized the baseline characteristics of the participants and major findings. The CAC was the first child cohort study in Southwest China that has used an annual multimodal assessment approach to prospectively address research questions on the associations between nutritional factors and later growth in children. The subjects would be followed up from the age of 6–8 till the age of 15, with follow-up assessments of anthropometry and puberty status every year and of diet and physical activity every 2 years.

To date, more than 10 child cohort studies have been launched in China. Each of those existing cohorts focused on different exposures or outcomes, starting their recruitment at different childhood periods and following up their participants in different time frames. For example, some child cohorts have examined the impacts of environmental factors [e.g., lead exposure and micronutrient deficiency (41, 42), phthalate exposure (43), early life feeding practice (44), and eating behaviors (45)] on children's cognitive and behavior outcomes [e.g., China Jintan Child Cohort Study (41, 42), Taiwan Maternal and Infant Cohort Study (43)], adiposity [e.g., Jiaxing Birth Cohort (44), Studying Impact of Nutrition on Growth (13), Childhood Obesity Study in China Mega-cities (45)], and physical, psychological, behavioral and sexual development [e.g., China-Anhui Birth Cohort study (17)]. Among them, only the China-Anhui Birth Cohort study has considered puberty timing as one of their outcomes. Although nutrition was also considered an exposure in that study, it did not specifically focus on nutritional factors as their main exposures. Moreover, it did not provide repeated and detailed exposure assessments across the entire pubertal period. Therefore, the capacity of estimating the longitudinal associations between dietary intake, body composition, and timing of puberty in that cohort was not comparable with that in the CAC study. Compared to the aforementioned child cohorts, a major advantage of the CAC study was the frequent follow-ups and detailed assessments of socio-demographic characteristics, nutrition, lifestyles, anthropometry, and pubertal development across the entire pubertal period (i.e., before, during, and after the pubertal period). In addition, information on characteristics of the participants' parents was also frequently collected. Furthermore, the CAC dataset was of high quality since all the interviews and examinations have been conducted by trained investigators. Due to the abundance of data and the long duration of follow-up, the temporal changes in children's body composition and processes of pubertal development could be tracked. Furthermore, the prospective associations between nutritional factors and the gradual changes in growth during childhood would be able to be determined. Despite the lack of comparable data from Southwest China, only minor differences were found in socio-demographic characteristics (characterized by household income and maternal/paternal education level) between participants in this study and those in the existing studies conducted in Sichuan (46) or Chongqing (47, 48), which might ensure generalizability of our findings. The results of baseline and the first follow-up data indicated that the CAC study would be a strong evidence-based platform for public health research.

Some limitations should be mentioned. First, attrition is a concern. Although we have made efforts to maintain the participants in the cohort, in common with most child cohort studies, the response rate has decreased over time. Until April 2021, 18.1% of the CAC participants were lost to the first follow-up, especially those with lower parental education and family income levels, which may result in the selection of a more “health conscious” study sample by the end of the follow-ups. It required more administrative efforts to partner with schools to enhance children's participation. Second, objective measures of physical activity such as pedometer are not used in the CAC study. However, the physical activity questionnaire adopted in the study has been validated and widely used in the literature (24, 49). Furthermore, serum or plasma biomarkers for vitamin C, calcium, iron, zinc, or cholesterol would have been preferable to verify the nutritional status of children. However, the inclusion of blood sampling is difficult, especially in most large observational studies of children. Finally, due to a focus on the period of middle childhood in the CAC study, we could not provide detailed data on early childhood. However, some important early-life exposures (i.e., children's birth weight, exclusive breast-feeding duration, and timing of complementary feeding) are collected in our study.

In future work, we hope to broaden both breadth and depth of measures in our study. We are also planning to include new domains in the CAC study, such as genetic factors, environmental exposures, and some biomarkers in at least one sub-cohort, although impossible for the entire cohort due to limited financial support. With these comprehensive and detailed data, we would be able to examine interactions between genetic and nutritional factors and their influences on children's growth and the underlying mechanisms. Furthermore, if funding comes through, we also hope to follow up the participants in the sub-cohort(s) until their adulthood.

## Data Availability Statement

The raw data supporting the conclusions of this article will be made available by the authors, without undue reservation.

## Ethics Statement

The studies involving human participants were reviewed and approved by the Ethics Committee of Sichuan University. Written informed consent to participate in this study was provided by the participants' legal guardian/next of kin.

## Author Contributions

GC conceived and supervised the project. RD performed the statistical analysis and wrote the manuscript. RD, XW, and SS took part in the data collection. LZ, JX, and LL coordinated the study centers and revised the manuscript. All authors contributed substantially to the work and approved the final version of the manuscript.

## Funding

This research was funded by a research grant from the New Century Excellent Talents in University Program (NCET-12-0377), Sichuan Outstanding Young Scholars Foundation (2014JQ0005), and the National Natural Science Foundation of China (81673158).

## Author Disclaimer

Frontiers Media SA remains neutral with regard to jurisdictional claims in published maps and institutional affiliations.

## Conflict of Interest

The authors declare that the research was conducted in the absence of any commercial or financial relationships that could be construed as a potential conflict of interest.

## Publisher's Note

All claims expressed in this article are solely those of the authors and do not necessarily represent those of their affiliated organizations, or those of the publisher, the editors and the reviewers. Any product that may be evaluated in this article, or claim that may be made by its manufacturer, is not guaranteed or endorsed by the publisher.

## References

[1] Bass R Eneli I. Severe childhood obesity: an under-recognised and growing health problem. Postgrad Med J. (2015) 91:639–45. 10.1136/postgradmedj-2014-133033 26338983

[2] Wang H Xue H Du S Zhang J Wang Y Zhang B. Time trends and factors in body mass index and obesity among children in China: 1997-2011. Int J Obes (Lond). (2017) 41:964–70. 10.1038/ijo.2017.53 28239162 PMC5890802

[3] Jia P Xue H Zhang J Wang Y. Time trend and demographic and geographic disparities in childhood obesity prevalence in china-evidence from twenty years of longitudinal data. Int J Environ Res Public Health. (2017) 14:369. 10.3390/ijerph14040369 28362361 PMC5409570

[4] Ma HM Chen SK Chen RM Zhu C Xiong F Li T. Pubertal development timing in urban Chinese boys. Int J Androl. (2011) 34:e435–45. 10.1111/j.1365-2605.2011.01173.x 21658069

[5] Ma HM Du ML Luo XP Chen SK Liu L Chen RM. Onset of breast and pubic hair development and menses in urban chinese girls. Pediatrics. (2009) 124:e269–77. 10.1542/peds.2008-2638 19651567

[6] Brisbois TD Farmer AP McCargar LJ. Early markers of adult obesity: a review. Obes Rev. (2012) 13:347–67. 10.1111/j.1467-789X.2011.00965.x 22171945 PMC3531624

[7] Umer A Kelley GA Cottrell LE Giacobbi P Jr Innes KE Lilly CL. Childhood obesity and adult cardiovascular disease risk factors: a systematic review with meta-analysis. BMC public health. (2017) 17:683. 10.1186/s12889-017-4691-z 28851330 PMC5575877

[8] Bodicoat DH Schoemaker MJ Jones ME McFadden E Griffin J Ashworth A. Timing of pubertal stages and breast cancer risk: the Breakthrough Generations Study. Breast Cancer Res. (2014) 16:R18. 10.1186/bcr3613 24495528 PMC3978643

[9] Popkin BM. Synthesis and implications: China's nutrition transition in the context of changes across other low- and middle-income countries. Obes Rev. (2014)15:60–7. 10.1111/obr.12120 24341759 PMC3869101

[10] Li W Liu Q Deng X Chen Y Liu S Story M. Association between obesity and puberty timing: a systematic review and meta-analysis. Int J Environ Res Public Health. (2017) 14:1266. 10.3390/ijerph14101266 29064384 PMC5664767

[11] Kroke A Manz F Kersting M Remer T Sichert-Hellert W Alexy U. The DONALD study. History, current status and future perspectives. Eur J Nutr. (2004) 43:45–54. 10.1007/s00394-004-0445-7 14991269

[12] Potischman N Fang L Hao L Bailey RR Berrigan D Berry RJ. The Chinese children and families cohort study: the nutrition, physical activity, and ultraviolet radiation data collection. Nutr Today. (2018) 53:104–14. 10.1097/NT.0000000000000275 29930434 PMC5999376

[13] Lee A Cheung CK Lo K Keung VM Mui LW Tam WWS. Studying Impact of Nutrition on Growth (SING): a prospective cohort for comparing the health outcomes of young children with the dietary quality score. BMJ Open. (2017) 7:e018380. 10.1136/bmjopen-2017-018380 29122800 PMC5695350

[14] Ahrens W Bammann K de Henauw S Halford J Palou A Pigeot I. Understanding and preventing childhood obesity and related disorders–IDEFICS: a European multilevel epidemiological approach. Nutr Metab Cardiovasc Dis. (2006) 16:302–8. 10.1016/j.numecd.2006.01.011 16679223

[15] Kooijman MN Kruithof CJ van Duijn CM Duijts L Franco OH van Ijzendoorn MH. The Generation R Study: design and cohort update 2017. Eur J Epidemiol. (2016) 31:1243–64. 10.1007/s10654-016-0224-9 28070760 PMC5233749

[16] Moreno LA De Henauw S González-Gross M Kersting M Molnár D Gottrand F. Design and implementation of the Healthy Lifestyle in Europe by Nutrition in Adolescence Cross-Sectional Study. Int J Obes (Lond). (2008) 32:S4–S11. 10.1038/ijo.2008.177 19011652

[17] Tao FB Hao JH Huang K Su PY Cheng DJ Xing XY. Cohort profile: the china-anhui birth cohort study. Int J Epidemiol. (2013) 42:709–21. 10.1093/ije/dys085 22729236

[18] Victora CG Hallal PC Araújo CL Menezes AM Wells JC Barros FC. Cohort profile: the 1993 Pelotas (Brazil) birth cohort study. Int J Epidemiol. (2008) 37:704–9. 10.1093/ije/dym177 17846051

[19] Gonçalves H Assunção MC Wehrmeister FC Oliveira IO Barros FC Victora CG. Cohort profile update: The 1993 Pelotas (Brazil) birth cohort follow-up visits in adolescence. Int J Epidemiol. (2014) 43:1082–8. 10.1093/ije/dyu077 24729426 PMC4121560

[20] Zhou X Xue H Duan R Liu Y Zhang L Harvey L. The cross-sectional association of energy intake and dietary energy density with body composition of children in Southwest China. Nutrients. (2015) 7:5396–412. 10.3390/nu7075228 26151177 PMC4517005

[21] Cheng G Duan RN Kranz S Liu Y Xue HM. Development of a dietary index to assess overall diet quality for chinese children and adolescents: The Chinese children dietary index. J Acad Nutr Diet. (2014) 116:608–17. 10.1016/j.jand.2015.11.010 26825477

[22] Yang YX He M Pan XC. China Food Composition. 2nd ed. Peking University Medical Press. (2004).

[23] Duan R Liu Y Xue H Yang M Cheng G. [Cross-sectional association between overall diet quality and overweight/obesity among children and adolescents in Chengdu]. Zhonghua Liu Xing Bing Xue Za Zhi. (2014) 35:994–8. 25492138

[24] Huang YJ Wong SH Salmon J. Reliability and validity of the modified Chinese version of the Children's Leisure Activities Study Survey (CLASS) questionnaire in assessing physical activity among Hong Kong children. Pediatr Exerc Sci. (2009) 21:339–53. 10.1123/pes.21.3.339 19827457

[25] Tanner JM. Normal growth and techniques of growth assessment. Clin Endocrinol Metab. (1986) 15:411–51. 10.1016/S0300-595X(86)80005-6 3533329

[26] National Bureau of Statistics of China. (2021). Available online at: http://data.stats.gov.cn/easyquery.htm?cn=C01. National Data

[27] Liu Y Duan R Xue H Yang M Zhou X Cheng G. [Overweight and obesity status among children and adolescents aged 7-15 years old in Chengdu]. Chinese Journal of School Health. (2014) 35:1191–3.

[28] Yang MZ Xue HM Pan J Libuda L Muckelbauer R Yang M. High protein intake along with paternal part-time employment is associated with higher body fat mass among girls from South China. Eur J Nutr. (2018) 57:1845–54. 10.1007/s00394-017-1468-1 28536743

[29] Chen Y Liu Y Xue H Bao Y Luo J Tian G. [Association between dietary calcium/dairy intakes and overweight/obesity]. Wei Sheng Yan Jiu. (2016) 45:402–8. 27459802

[30] Li DT Chen MX Xue HM Li Y Tian G Gong YH. [Association between dietary patterns and overweight/obesity among school-aged children in Chengdu]. Modern Preventive Medicine. (2018) 45:4070–3.

[31] Duan R Xue H Liu Y Yang M Cheng G. [Cross-sectional association between eating behaviors and overweight/obesity among children and adolescents in Chengdu city]. Wei Sheng Yan Jiu. (2015) 44:576–80. 26454954

[32] Zhai J Xue H Luo J Zhang L Cheng G. Associations between socioeconomic status and overweight among urban children aged 7-12 years in Chengdu, southwest China. Asia Pac J Clin Nutr. (2018) 27:617–23. 10.6133/apjcn.022017.12 29737809

[33] Yang M Wang D Hu JX Liu Y Duan R Xue H. [Pubertal development among children in Chengdu and associated family factors]. Chin J Sch Health. (2015) 36:226–30, 35.

[34] Tian G Liu Y Xue HM Luo J Chen YR Bao YX. [Dietary fiber and pubertal development among children and adolescents–a cross-sectional study in Chengdu, Sichuan]. Sichuan da xue xue bao Yi xue ban = Journal of Sichuan University Medical science edition. (2016) 47:244–7. 27263303

[35] Liu Y Duan R Xue H Yang M Cheng G. [Cross-sectional association between consumption of grain/tubers and pubertal development among children and adolescents in Chengdu City]. Wei Sheng Yan Jiu. (2015) 44:49–52. 25958635

[36] Luo J Yang MZ Duan RN Tian G Bao YX Chen YR. [Consumptions of Meat and dairy products, zinc intake and pubertal development in adolescents in Chengdu]. Sichuan da xue xue bao Yi xue ban = Journal of Sichuan University Medical science edition. (2015) 46:726–31. 26619544

[37] Duan R Qiao T Chen Y Chen M Xue H Zhou X. The overall diet quality in childhood is prospectively associated with the timing of puberty. Eur JNutr. (2020). 10.1007/s00394-020-02425-8 PMC8275527 33140158

[38] Liu Y Yang M Duan R Xue H Cheng G. [Dietary behaviors among children and adolescents in Chengdu and its association with pubertal development]. Chin J Sch Health. (2015) 36:1126–9.

[39] Xue H Yang M Duan R Liu Y Cheng G Zhang X. [Relationship between family socio-economic factors and sedentary behaviors among children and adolescents in Chengdu city]. Chin J Sch Health. (2015) 36:47–50, 3.

[40] Xue H Tian G Duan R Quan L Zhao L Yang M. Sedentary behavior is independently related to fat mass among children and adolescents in South China. Nutrients. (2016) 8. 10.3390/nu8110667 PMC5133055 27792134

[41] Liu J McCauley LA Zhao Y Zhang H Pinto-Martin J. Cohort Profile: The China Jintan child cohort study. Int J Epidemiol. (2010) 39:668–74. 10.1093/ije/dyp205 19433517 PMC2912482

[42] Liu J Cao S Chen Z Raine A Hanlon A Ai Y. Cohort Profile Update: The China Jintan Child Cohort Study. Int J Epidemiol. (2015) 44:1548. 10.1093/ije/dyv119 26323725 PMC4707195

[43] Wu CF Chen HM Sun CW Chen ML Hsieh CJ Wang SL. Cohort Profile: The Taiwan Maternal and Infant Cohort Study (TMICS) of phthalate exposure and health risk assessment. Int J Epidemiol. (2018) 47:1047. 10.1093/ije/dyy067 29718277

[44] Zheng JS Liu H Zhao YM Li J Chen Y Zhu S. Complementary feeding and childhood adiposity in preschool-aged children in a large Chinese cohort. J Pediatr. (2015) 166:326–31.e2. 10.1016/j.jpeds.2014.11.010 25491091

[45] Jia P Li M Xue H Lu L Xu F Wang Y. School environment and policies, child eating behavior and overweight/obesity in urban China: the childhood obesity study in China megacities. Int J Obes (Lond). (2017) 41:813–9. 10.1038/ijo.2017.2 28074059

[46] Zhang J Mou Y Liao J Xiong H Duan Z Huang Y. Uptake of maternal care and childhood immunization among ethnic minority and Han populations in Sichuan province: a study based on the 2003, 2008 and 2013 health service surveys. BMC Pregnancy Childbirth. (2019) 19:250. 10.1186/s12884-019-2371-y 31311501 PMC6636102

[47] Li W Liu Q Chen Y Yang B Huang X Li Y. Effects of indoor environment and lifestyle on respiratory health of children in Chongqing, China. J Thorac Dis. (2020) 12:6327–41. 10.21037/jtd.2020.03.102 33209472 PMC7656398

[48] Yang B Ostbye T Huang X Li Y Fang B Wang H. Maternal age at menarche and pubertal timing in boys and girls: a cohort study from Chongqing, China. J Adolesc Health. (2021) 68:508–16. 10.1016/j.jadohealth.2020.06.036 32798100

[49] Matthews CE Shu XO Yang G Jin F Ainsworth BE Liu D. Reproducibility and validity of the Shanghai Women's Health Study physical activity questionnaire. Am J Epidemiol. (2003) 158:1114–22. 10.1093/aje/kwg255 14630608

